# Fatal Necrotizing Soft Tissue Infection Following Continuous Glucose Monitoring in a Patient with Type 1 Diabetes: A Case Report and Literature Review

**DOI:** 10.3390/diseases14040124

**Published:** 2026-03-31

**Authors:** Constantin Popazu, Cristiana Voineag, Ionica Grigore, Cristina Șerban, Mădălin Guliciuc, Dragoș Voicu, Alexandra Toma

**Affiliations:** 1 Faculty of Medicine and Pharmacy, Research Centre in the Medical-Pharmaceutical Field, “Dunărea de Jos” University of Galați, 800201 Galați, Romania; 2 County Emergency Clinical Hospital of Brăila, 810325 Brăila, Romania; 3 “Sf. Apostol Andrei” County Emergency Clinical Hospital Galați, 800578 Galați, Romania

**Keywords:** continuous glucose monitoring, necrotizing soft tissue infection, type 1 diabetes mellitus, device-associated infection, skin barrier disruption, sepsis, case report

## Abstract

Background: Continuous glucose monitoring (CGM) systems have significantly improved glycemic management in patients with type 1 diabetes mellitus and are generally considered safe. However, transcutaneous sensor insertion disrupts the skin barrier and, in susceptible individuals, may contribute to infectious complications. Severe soft tissue infections occurring in temporal association with CGM use are exceedingly rare. Case Presentation: We report a fatal case of necrotizing soft tissue infection in a 54-year-old male with long-standing type 1 diabetes mellitus occurring in temporal association with CGM use. The patient initially developed localized inflammation at a prior sensor insertion site that failed to fully resolve. Over subsequent weeks, he experienced progressive systemic symptoms and worsening local findings, culminating in advanced necrotizing infection. Despite emergency surgical debridement, broad-spectrum antimicrobial therapy, and intensive care support, the clinical course was complicated by septic shock and multiorgan failure, resulting in death. Discussion: This case highlights the role of patient-specific vulnerability, persistent insertion-site inflammation, and delayed clinical recognition in the progression from localized skin changes to life-threatening infection. Importantly, this report does not establish a direct causal relationship between CGM use and necrotizing soft tissue infection but underscores the need for heightened vigilance in high-risk individuals. Conclusions: Although CGM systems have a favorable safety profile, careful inspection of insertion sites, avoidance of sensor reapplication over incompletely healed tissue, and early evaluation of persistent or progressive symptoms are essential to minimize the risk of severe outcomes in susceptible patients.

## 1. Introduction

Continuous glucose monitoring (CGM) systems have transformed the management of diabetes mellitus, particularly in patients with type 1 diabetes, by enabling continuous assessment of interstitial glucose levels, reducing glycemic variability, and improving overall quality of life [1,2]. Their widespread adoption in both outpatient and inpatient settings reflects robust evidence supporting improved glycemic outcomes, reduced hypoglycemic events, and enhanced patient engagement in self-management. As CGM technology becomes increasingly integrated into routine clinical practice, the safety profile of these devices remains a critical consideration.

CGM devices require transcutaneous sensor insertion, which inherently disrupts the integrity of the skin barrier [3,4]. As the skin represents a primary defense against microbial invasion, even small breaches may facilitate bacterial adherence and colonization. Repeated sensor insertions, prolonged wear time, occlusion, and local microtrauma further contribute to alterations in the cutaneous microenvironment that may promote infection, particularly in susceptible individuals [5,6].

Most adverse skin reactions associated with CGM systems are mild and self-limiting, typically manifesting as irritant or allergic contact dermatitis, localized erythema, pruritus, and minor insertion-site trauma [2,5]. These events are generally managed conservatively and rarely require discontinuation of therapy. However, increasing device utilization has been accompanied by a broader spectrum of reported skin complications, ranging from superficial bacterial colonization and localized cellulitis to device-associated infections that may serve as portals of entry for invasive pathogens [3,6].

Although severe infections related to CGM use are uncommon, sporadic case reports have documented serious complications, including abscess formation and necrotizing soft tissue infections, following sensor insertion [7,8]. These reports raise concern that, under certain conditions, persistent local inflammation, delayed healing, inadequate site monitoring, or premature reapplication of sensors may enable progression from superficial infection to deep fascial involvement. Device-related skin disruption may therefore represent an underrecognized risk factor for severe infection in select patient populations.

Patients with diabetes mellitus are particularly vulnerable to skin and soft tissue infections due to a combination of impaired innate and adaptive immune responses, microvascular dysfunction, peripheral neuropathy, and delayed wound healing [9,10,11]. These pathophysiological alterations not only increase susceptibility to infection but also contribute to more aggressive disease courses and poorer outcomes. Diabetes has been consistently identified as an independent risk factor for rapid progression and increased mortality in necrotizing soft tissue infections [12,13,14].

Early clinical manifestations of necrotizing soft tissue infections are often nonspecific and may mimic benign conditions such as cellulitis, resulting in delayed diagnosis and advanced disease at presentation [13,15]. The potential for negative microbiological cultures, particularly following empirical antibiotic therapy or in polymicrobial infections, further complicates timely recognition. Consequently, a high index of suspicion is required when evaluating persistent or progressive skin changes in diabetic patients, especially in the context of medical device use.

Despite the growing body of literature on dermatologic complications of CGM systems, reports of life-threatening infections remain exceedingly rare, and fatal outcomes have not been previously documented for several modern devices.

Importantly, although continuous glucose monitoring systems are widely regarded as safe and are associated predominantly with mild and self-limited cutaneous reactions, their use involves repeated transcutaneous sensor insertion, which inherently disrupts the integrity of the skin barrier. In most individuals, such disruption does not result in clinically significant complications; however, in susceptible patients, even minor or repetitive breaches of the skin may facilitate microbial colonization and persistence, particularly when local inflammation fails to fully resolve.

The risk of infection following CGM insertion appears to be influenced predominantly by patient-related factors rather than by intrinsic device-related hazards. Long-standing diabetes mellitus is associated with impaired innate and adaptive immune responses, microvascular dysfunction, altered inflammatory signaling, and delayed wound healing, all of which may compromise the host’s ability to contain localized skin infections. Additional factors such as glycemic variability, peripheral neuropathy, repeated mechanical microtrauma, occlusion at insertion sites, and reduced perception of early symptoms may further contribute to delayed recognition and progression of infection.

In this context, persistent insertion-site erythema, induration, or tenderness may represent not merely a benign local reaction but an early manifestation of a more complex pathological process. The distinction between irritant or allergic reactions and evolving infection can be clinically challenging, particularly in outpatient settings, where early signs may be subtle and systemic manifestations absent. Consequently, delayed clinical assessment or premature reapplication of CGM sensors over incompletely healed tissue may increase the likelihood of progression from superficial skin involvement to deeper soft tissue infection.

It is therefore critical to emphasize that CGM use should not be interpreted as a direct causal trigger of severe soft tissue infection. Rather, CGM-related skin disruption should be regarded as a potential contributory factor within a multifactorial clinical framework that includes individual susceptibility, local tissue vulnerability, and delays in diagnosis or intervention. Severe infectious complications reported in temporal association with CGM use appear to be exceedingly rare and likely reflect an interaction between patient-specific risk factors and localized skin barrier compromise, rather than a generalized safety concern related to CGM technology itself.

Against this background, the present case is reported to raise awareness of a rare but potentially catastrophic outcome occurring in temporal association with CGM use in a patient with type 1 diabetes mellitus. By contextualizing this event within the broader literature on CGM-associated cutaneous complications and diabetes-related infection risk, this report aims to highlight the importance of careful insertion-site surveillance, avoidance of sensor reapplication over unresolved inflammatory lesions, and early clinical evaluation of persistent or progressive local symptoms in high-risk patients.

In this context, we report a fatal case of necrotizing soft tissue infection in a patient with type 1 diabetes following the use of a Dexcom ONE™ continuous glucose monitoring system. To our knowledge, this represents the first reported fatal outcome associated with this device. This case highlights the potential for catastrophic infectious complications arising from CGM sensor sites and underscores the critical importance of early recognition, avoidance of device reapplication over incompletely healed tissue, and structured follow-up in high-risk patients.

## 2. Case Presentation

A 54-year-old male with a history of type 1 diabetes mellitus presented with a fulminant necrotizing soft tissue infection following the use of a continuous glucose monitoring (CGM) system. The patient had no documented history of immunosuppressive therapy, malignancy, or chronic inflammatory disease.

The patient had a long-standing history of type 1 diabetes mellitus, with the first documented presentation to a specialized diabetes outpatient clinic in 2013, at which time insulin therapy with human insulin (Humulin) was initiated. Based on the available medical records, the estimated duration of diabetes at the time of the infectious event was approximately 11 years. Over the course of the disease, diabetes-related chronic complications gradually developed, including diabetic nephropathy.

Longitudinal data regarding glycemic control were incompletely documented. Nevertheless, the most recent available metabolic assessment prior to the acute presentation indicated acceptable glycemic control, with a reported glycated hemoglobin (HbA1c) value of 6.9%. No serial HbA1c measurements were available to reliably assess long-term glycemic trends or variability.

Diabetes management consisted of multiple daily insulin injections. According to available clinical documentation, insulin was administered at anatomical sites distinct from the right upper arm, and no local inflammatory reactions, skin breakdown, or infectious complications were reported at insulin injection sites throughout the course of treatment.

Continuous glucose monitoring was initiated for the first time on 09 June 2023, representing the patient’s initial exposure to CGM technology. According to patient-reported information documented in the medical record, routine skin hygiene measures were followed prior to sensor insertion. There was no documentation of improper handling, insertion errors, prolonged sensor wear beyond recommended intervals, or other deviations from standard device use.

In July 2024, a CGM sensor was applied to the right upper arm as part of routine diabetes management. Within days of insertion, the patient developed localized erythema, swelling, tenderness, and warmth at the sensor site, consistent with a diagnosis of cellulitis. The sensor was removed, and the patient received oral systemic antibiotic therapy in the outpatient setting. No imaging studies or microbiological cultures were obtained at that time. Although partial clinical improvement was described, complete resolution of local signs was not achieved, and residual induration and erythema persisted at the insertion site.

In October 2024, continuous glucose monitoring therapy was resumed with placement of a new sensor on the contralateral (left) upper arm, without immediate local complications at the new insertion site. Over the following weeks, the patient experienced progressive systemic symptoms, including persistent fatigue, malaise, intermittent low-grade fever, and gradually worsening discomfort and swelling in the right upper arm, corresponding to the initial sensor site. These symptoms were not evaluated medically, contributing to a significant delay in diagnosis.

In January 2025, approximately three months after reinitiation of CGM therapy, the patient presented to the emergency department with marked deterioration of his general condition, including severe asthenia, diffuse myalgias, and features of systemic illness. On admission, laboratory investigations revealed extreme leukocytosis (44,000/µL), elevated inflammatory markers, metabolic acidosis, and acute kidney injury, raising concern for sepsis. Prior to surgical admission, the patient required hospitalization in a nephrology department for acute kidney injury, interpreted in the context of underlying diabetic nephropathy. During this period, progressive systemic symptoms and worsening local inflammatory changes involving the right upper arm were noted, prompting further clinical evaluation. Given the rapid deterioration of the patient’s general condition and the progression of local findings, surgical consultation was requested, and the patient was subsequently transferred for definitive management of a suspected necrotizing soft tissue infection.

**Figure 1:**
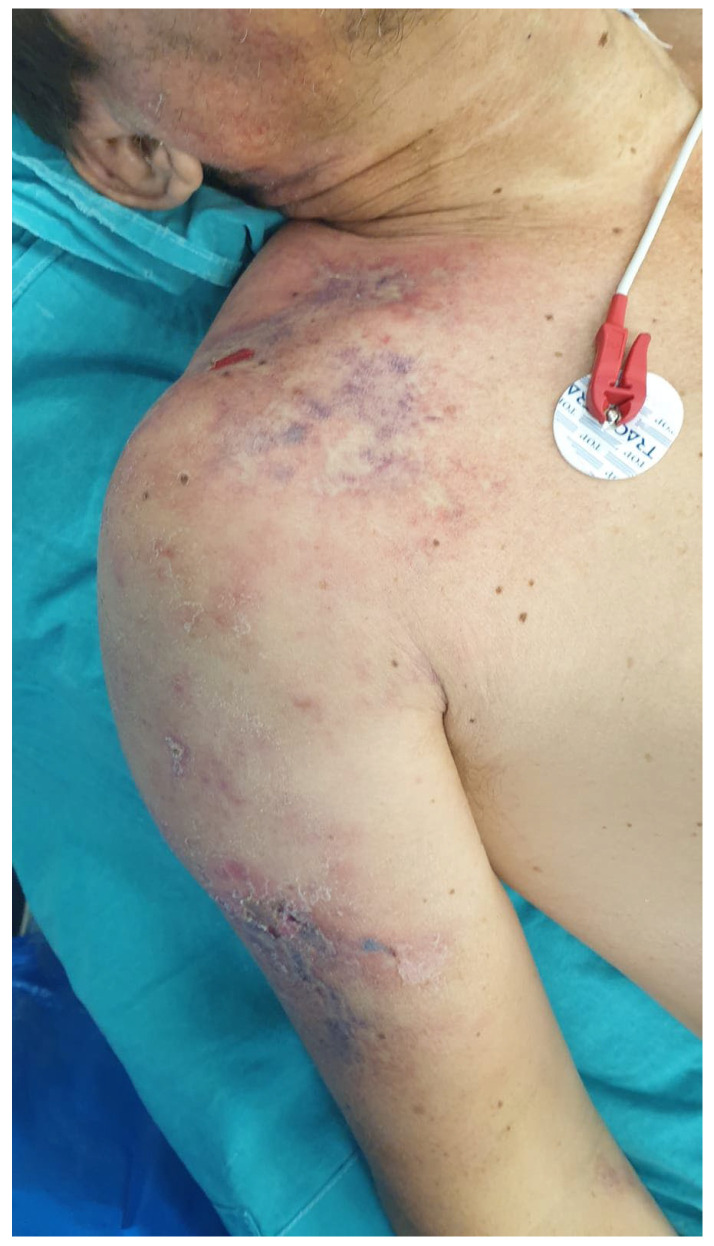
Clinical findings of the affected area.

Physical examination demonstrated extensive soft tissue edema involving the proximal right upper arm, extending to the shoulder, supraclavicular region, and upper right hemithorax. The affected area was warm, tense, and markedly painful, with pain disproportionate to physical findings. Areas of skin discoloration and evolving necrosis were noted (Figure 1). Vital signs revealed tachycardia and low-grade pyrexia, further supporting the suspicion of a necrotizing soft tissue infection.

**Figure 2:**
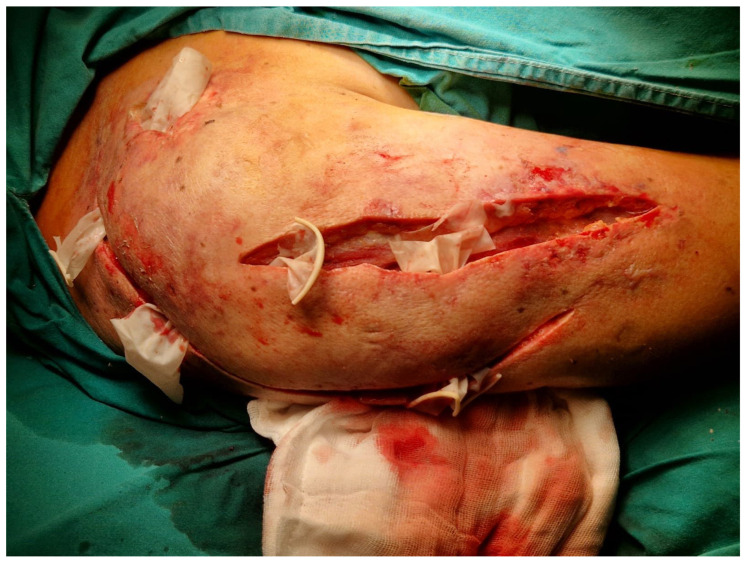
Intraoperative aspect of the affected area.

Given the rapid clinical progression and concerning examination findings, emergent surgical exploration was undertaken without delay. Intraoperative findings revealed widespread necrosis of the superficial and deep fascial layers, purulent collections tracking along musculofascial planes, and extensive tissue devitalization consistent with necrotizing soft tissue infection (Figure 2). Surgical management consisted of radical debridement of all nonviable tissue, multiple longitudinal incisions to decompress the affected compartments, extensive irrigation, and open drainage.

Broad-spectrum intravenous antimicrobial therapy was initiated immediately following surgery, and the patient was transferred to the intensive care unit for advanced supportive care. His postoperative course was complicated by septic shock, necessitating vasopressor support, mechanical ventilation, and continuous renal replacement therapy. Despite aggressive surgical intervention and maximal critical care support, the patient developed progressive multiorgan failure and died on postoperative day five.

## 3. Discussion

This case illustrates how device-associated skin disruption may contribute to severe infectious complications in susceptible patients. Continuous transcutaneous devices, including CGM systems, create repeated breaches of the skin barrier, which may facilitate microbial colonization and persistence when local inflammation fails to resolve [3,4,6]. Occlusion, microtrauma related to sensor insertion, prolonged wear time, and exposure to adhesive materials may further alter the cutaneous microenvironment, promoting bacterial adherence and impaired local clearance [5,6]. In patients with diabetes, these factors may allow a clinically indolent process to evolve silently into deep soft tissue infection before overt systemic manifestations become apparent [10,11].

Importantly, the present case does not establish a direct causal relationship between continuous glucose monitoring use and the development of necrotizing soft tissue infection. As a single observational report, it cannot determine whether CGM use directly triggered the infectious process. Instead, CGM-related skin disruption should be interpreted as a potential contributory factor within a complex, multifactorial clinical scenario.

The temporal association between sensor insertion and infection onset must therefore be approached with caution, particularly in a patient population characterized by impaired wound healing and altered immune responses. Overinterpretation of such associations risks attributing causality where only coexistence or facilitation may be present. From a scientific and clinical perspective, it is more appropriate to consider CGM use as one element within a broader interplay of patient-specific vulnerabilities, local tissue susceptibility, and delayed clinical recognition, rather than as a direct etiological agent.

Diabetes mellitus is a well-established risk factor for skin and soft tissue infections and is independently associated with more aggressive disease courses and poorer outcomes [10,11,12]. Impaired innate immune responses, including defective neutrophil chemotaxis and phagocytosis, combined with microvascular dysfunction and tissue ischemia, markedly reduce the host’s ability to contain localized infections [11,12,13]. These pathophysiological mechanisms contribute to the rapid progression, increased severity, and disproportionately high mortality observed in necrotizing soft tissue infections among diabetic patients [12,13,14].

Early clinical manifestations of necrotizing soft tissue infections are frequently nonspecific and may resemble benign conditions such as uncomplicated cellulitis, which contributes to delayed diagnosis [13,15]. Pain disproportionate to physical findings, progressive edema, and subtle systemic symptoms may initially be overlooked, particularly in outpatient settings. Once deep fascial involvement is established, the therapeutic window narrows considerably, and outcomes are largely determined by the extent of infection at presentation rather than the promptness of intervention thereafter [14,15]. This underscores the importance of maintaining a low threshold for surgical consultation in high-risk patients, including those with diabetes and medical device–associated skin changes.

Microbiological cultures may be negative in advanced necrotizing soft tissue infections, particularly following prior antibiotic exposure or in polymicrobial or fastidious infections [14,16]. Consequently, the absence of microbiological confirmation should not delay definitive management when clinical and intraoperative findings are suggestive of necrotizing infection. In such cases, diagnosis remains primarily clinical and surgical, supported by imaging and intraoperative assessment rather than laboratory or microbiological parameters alone [13,17].

While CGM systems are generally safe and well tolerated, this case highlights the potential risks associated with reapplication of sensors in the context of unresolved local inflammation. Persistent insertion-site changes may serve as a reservoir for ongoing microbial colonization, even in the absence of overt clinical infection [3,6]. Repeated sensor placement over anatomically proximate or previously affected areas may further increase the risk of bacterial seeding and progression to deep tissue involvement [4,6]. Careful inspection of prior insertion sites, adequate healing intervals, and avoidance of reapplication over incompletely healed tissue should therefore be emphasized, particularly in patients with diabetes or other predisposing conditions [6,9].

Repeated emphasis on CGM sensor reapplication in this case should not be interpreted as indicating a generalized device-related hazard. Continuous glucose monitoring systems have demonstrated a favorable safety profile across large and diverse patient populations. The occurrence of severe infection in this context more likely reflects individual susceptibility and contextual clinical factors rather than an intrinsic risk associated with the device itself.

Factors such as unresolved local inflammation, impaired tissue repair, delayed symptom recognition, and patient-specific vulnerability appear to play a decisive role in determining risk. Distinguishing between individual risk and device-related risk is essential to avoid misinterpretation of isolated cases and to prevent undue concern regarding widely used and clinically beneficial technologies.

Published reports of CGM-associated infections have predominantly described localized or reversible complications, including cellulitis and subcutaneous abscess formation [7,8]. Only isolated cases of necrotizing soft tissue infection following CGM use have been documented, most of which were associated with early recognition and favorable outcomes after prompt surgical intervention [7]. In contrast, the present case demonstrates how delayed clinical recognition and persistent local inflammation may permit progression to catastrophic infection, despite subsequent aggressive management.

Severe infections associated with continuous glucose monitoring systems appear to be exceedingly rare, based on available postmarketing surveillance data, registry reports, and published case literature. The vast majority of reported cutaneous complications related to CGM use consist of mild and reversible conditions, such as irritant or allergic contact dermatitis, localized erythema, and superficial infections.

Reports of invasive soft tissue infection temporally associated with CGM insertion are limited to isolated case reports, and fatal outcomes are exceptional. This epidemiological context is essential for calibrating perceived risk and for preventing overgeneralization from single cases. The present report should therefore be interpreted as highlighting a rare but severe complication occurring under specific clinical circumstances, rather than as evidence of a broader safety concern related to CGM technology.

Several features distinguish this case from those previously reported. First, the necrotizing infection developed at a prior sensor insertion site where inflammation failed to fully resolve, suggesting a role for subclinical or chronic local infection. Second, despite reapplication of the CGM system at a contralateral site, infection progressed at the original location, underscoring the importance of monitoring all prior insertion sites rather than focusing exclusively on the most recent sensor placement. Finally, the fatal outcome observed contrasts with the generally favorable prognosis reported in earlier CGM-associated infections, highlighting the narrow therapeutic window once deep fascial involvement is established [7,8,14].

Distinguishing benign insertion-site reactions from early signs of infection remains clinically challenging, particularly in patients with diabetes. Mild erythema, pruritus, or localized discomfort are common and often self-limiting, which may contribute to normalization of early inflammatory signs. However, progressive pain, persistent erythema, induration, increasing edema, or the development of systemic symptoms should raise immediate concern for evolving infection.

In diabetic patients, peripheral neuropathy, altered pain perception, and impaired inflammatory responses may further obscure early warning signs, increasing the likelihood of delayed presentation. This diagnostic ambiguity underscores the importance of patient education, regular inspection of insertion sites, and a low threshold for medical evaluation when local symptoms fail to resolve or worsen over time.

From a clinical perspective, this case underscores the importance of comprehensive patient education regarding insertion-site hygiene, rotation protocols, and early warning signs of infection. Clinicians should maintain a high index of suspicion for deep soft tissue infection in diabetic patients presenting with unexplained pain, progressive edema, or systemic symptoms at or near CGM insertion sites [10,12,18,19,20]. Structured follow-up, standardized site surveillance, and avoidance of device reapplication in the presence of unresolved local inflammation are essential strategies to minimize the risk of severe outcomes as CGM use continues to expand [6,9,18].

## 4. Conclusions

This case reports a rare and fatal necrotizing soft tissue infection occurring in temporal association with continuous glucose monitoring use in a patient with type 1 diabetes mellitus. Although CGM systems are generally safe, transcutaneous sensor insertion may represent a contributory factor to severe infection in susceptible individuals when combined with patient-specific vulnerabilities and delayed clinical recognition.

Importantly, this report does not establish a direct causal relationship between CGM use and necrotizing soft tissue infection. Rather, it emphasizes the role of individual risk factors, persistent insertion-site inflammation, and delayed evaluation in the progression from localized skin changes to life-threatening infection. Careful inspection of insertion sites, avoidance of sensor reapplication over incompletely healed tissue, and early medical assessment of persistent or worsening symptoms are essential to minimize the risk of severe outcomes.

## Data Availability

Datasets used in this study are available upon request.

## Abbreviations

The following abbreviations are used in this manuscript:

CGM	Continuous Glucose Monitoring
NSTI	Necrotizing Soft Tissue Infection
T1DM	Type 1 Diabetes Mellitus
HbA1c	Hemoglobin A1c
ICU	Intensive Care Unit
CRRT	Continuous Renal Replacement Therapy
WBC	White Blood Cell count
MSSA	Methicillin-Sensitive *Staphylococcus* *aureus*
GAS	Group A *Streptococcus*
